# Californian Blastomycosis

**Published:** 1916-05

**Authors:** 


**Californian Blastomycosis.** Paul Campiche, of San Francisco, Rev. Med. de la Suisse Romande, Nov. 20, 1915, writes of the oidium coccidioides under this term, giving a hopeless prognosis, even with salvarsan. He considers the condition as limited to California and believes that European surgeons will never see it.

